# SLC38A2 provides proline and alanine to regulate postnatal bone mass accrual in mice

**DOI:** 10.3389/fphys.2022.992679

**Published:** 2022-09-23

**Authors:** Leyao Shen, Yilin Yu, Courtney M. Karner

**Affiliations:** ^1^ Department of Internal Medicine, Division of Nephrology, University of Texas Southwestern Medical Center, Dallas, TX, United States; ^2^ Charles and Jane Pak Center for Mineral Metabolism and Clinical Research, University of Texas Southwestern Medical Center, Dallas, TX, United States; ^3^ Department of Orthopaedic Surgery, Duke University School of Medicine, Durham, NC, United States

**Keywords:** proline, Alanine (Ala), Amino acid, Osteoblast (OB), bone

## Abstract

Amino acids have recently emerged as important regulators of osteoblast differentiation and bone formation. Osteoblasts require a continuous supply of amino acids to sustain biomass production to fuel cell proliferation, osteoblast differentiation and bone matrix production. We recently identified proline as an essential amino acid for bone development by fulfilling unique synthetic demands that are associated with osteoblast differentiation. Osteoblasts rely on the amino acid transporter SLC38A2 to provide proline to fuel endochondral ossification. Despite this, very little is known about the function or substrates of SLC38A2 during bone homeostasis. Here we demonstrate that the neutral amino acid transporter SLC38A2 is expressed in osteoblast lineage cells and provides proline and alanine to osteoblast lineage cells. Genetic ablation of SLC38A2 using *Prrx1Cre* results in decreased bone mass in both male and female mice due to a reduction in osteoblast numbers and bone forming activity. Decreased osteoblast numbers are attributed to impaired proliferation and osteogenic differentiation of skeletal stem and progenitor cells. Collectively, these data highlight the necessity of SLC38A2-mediated proline and alanine uptake during postnatal bone formation and bone homeostasis.

## Introduction

The mammalian skeleton is comprised of many bones which have diverse functions including support, protection of internal organs, mobility, endocrine signaling, mineral storage, and they are a site for red blood cell production (Guntur and Rosen, 2012; Long, 2012; Jagannathan-Bogdan and Zon, 2013; Salhotra et al., 2020). The individual bones are formed embryonically but continue to be maintained throughout life. Postnatal bone mass is regulated by the opposing activities of two cell types, bone forming osteoblasts and bone resorbing osteoclasts. Osteoblasts are responsible for the production of COL1A1 and other matricellular proteins that comprise the mineralized bone matrix. Any perturbation that reduces either osteoblast numbers or bone forming activity can result in declining bone mass and increased bone fragility, hall marks of human osteoporosis.

Osteoblasts differentiate from multipotent skeletal stem and progenitor cells (SSPC) that reside in the bone marrow and periosteum (Mizoguchi et al., 2014; Zhou et al., 2014; Bianco and Robey, 2015; Debnath et al., 2018). These SSPC can be targeted in the mouse using various CRE lines, including *Prxx1Cre* (Zhou et al., 2014; Ortinau et al., 2019). Osteoblast differentiation and bone formation is a well-orchestrated process. SSPC are proliferative before exiting the cell cycle as they undergo differentiation and increase bone matrix production (Pardee et al., 1978; Stein et al., 1990; Quarles et al., 1992; Stein and Lian, 1993). Proliferation, differentiation and protein synthesis increases the demand for nutrients like amino acids. Amino acids are essential for biomass production to support cell duplication and the production of osteoblast specific proteins and bone matrix. Consistent with this, the uptake of numerous amino acids including glutamine, proline and asparagine increases during osteoblast differentiation (Yu et al., 2019; Sharma et al., 2021; Shen et al., 2022). Genetic studies targeting either amino acid sensing pathways or transcription factors regulating amino acid uptake leads to reduced bone formation in mice (Elefteriou et al., 2006; Rached et al., 2010; Hu et al., 2020). Moreover, genetically targeting individual amino acid transporters inhibited bone development due to reduced osteoblast differentiation and decreased bone formation (Sharma et al., 2021; Shen et al., 2022). Thus, amino acid supply is critical for osteoblast endowment and bone mass regulation.

We recently identified proline as an essential amino acid for bone development by fulfilling unique synthetic demands that are associated with osteoblast differentiation (Shen et al., 2022). Proline consumption in osseous tissues has been under investigation for decades. Early studies characterized proline uptake in both bones and osteoblasts directly. These studies described proline uptake occurring primarily via System A but did not identify individual transporters mediating proline uptake (Finerman and Rosenberg, 1966; Adamson and Ingbar, 1967; Hahn et al., 1969; Yee, 1988). Consistent with these studies, we recently demonstrated the System A transporter SNAT2 (encoded by *Slc38a2* and denoted from now on as SLC38A2) is the primary proline transporter in embryonic osteoblasts (Shen et al., 2022). Genetic ablation of SLC38A2 in preosteoblasts using *Sp7Cre* prevented terminal osteoblast differentiation and inhibited both endochondral and intramembranous ossification. Whether SLC38A2 functions after birth during postnatal bone homeostasis was not evaluated. Interestingly, earlier studies indicated that proline transport characteristics differed in fetal and adult bones (Hahn et al., 1969). This suggests proline uptake may be mediated by distinct systems or transporters during embryonic bone development and postnatal bone homeostasis. However, very little is known about the proline transporter or transporters during bone homeostasis.

Here we demonstrate that SLC38A2 is the primary proline transporter in postnatal bones. Moreover, we demonstrate that SLC38A2 is a critical regulator of postnatal bone mass in mice. Using a genetic approach, we found SLC38A2 acts cell autonomously in osteoblast lineage cells to regulate SSPC proliferation, terminal osteoblast differentiation and bone matrix production. Mechanistically, we found that SLC38A2 provides both proline and alanine to regulate these processes. Collectively, these data highlight the essential nature of SLC38A2 to regulate postnatal bone homeostasis.

## Materials and methods

### Mouse strains

C57Bl/6J (RRID: IMSR_JAX:000664), *Rosa26* ^*Cas9*^ (RRID: IMSR_JAX:024858), *Rosa26* ^*FLP*^ (RRID: IMSR_JAX:003946) and *Prrx1Cre* (RRID: IMSR_JAX:005584) mouse strains were obtained from the Jackson Laboratory. *Slc38a2* ^*LacZ*^ (C57BL/6N-A<tm1Brd>Slc38a2<tm1a (KOMP)Wtsi>/Wtsi Ph) was purchased from the European Mouse Mutant Archive. To generate *Slc38a2* ^*flox*^, *Slc38a2* ^*LacZ*^ mice were crossed to *Rosa26* ^*FLP*^ to remove FRT-flanking LacZ cassette followed by a backcrossing with C57Bl/6J to remove *Rosa26* ^*FLP*^ allele. Mice were housed at 23°C on a 12-h light/dark cycle with free access to water and PicoLab Rodent Diet 20 (LabDiet #5053, St. Louis, MO). All mouse procedures were approved by the Animal Studies Committees at Duke University first and then the University of Texas Southwestern Medical Center.

### Mouse analysis

Micro computed tomography (VivaCT80, Scanco Medical AG) was used for three-dimensional reconstruction and analysis of bone parameters (threshold = 320) from 200 slices underneath the growth plate. Adult bones were fixed in 10% buffered formalin overnight at 4°C and scanned. Bones were then decalcified in 14% EDTA for 2 weeks at 4°C before processed for paraffin embedding and sectioned at 5 μ m using a Microtome (Leica RM2255). For OCN immunofluorescence staining, antigen retrieval was performed by soaking tissue sections in 10 mg/ml Proteinase K in PBS for 10 min at room temperature before blocking (1.5% goat serum in PBST) for 30 min. Sections were then incubated with anti-OCN (AB_1587337 diluted 1:250 in blocking solution) at 4°C overnight and then incubated with Alexa Fluor 568 goat anti-rabbit (AB_143157 at 1:250 dilution at room temperature for 30min. For dynamic histomorphometry, mice were injected with calcein (25 mg/kg) and alizarin red (75 mg/kg) intraperitoneally at 7 and 2 days prior to sacrifice respectively. Freshly isolated femurs were fixed in 4% PFA, cryoprotected in 30% sucrose then embedded in Cryomatrix for cryosectioning using cryojane. Dynamic histomorphometry parameters were quantified using ImageJ. For LacZ staining, isolated tissues were fixed (1% paraformaldehyde, 0.2% glutaraldehyde in PBS) on ice for 1 h, washed three times in 0.02% NP-40 in PBS and stained with 1 mg/ml X-Gal (5 mM K_3_Fe(CN)_6_, 5 mM K_4_Fe(CN)_6_, 2MM MgCl_2_, 0.01% Sodium Deoxycholate, 0.02% NP-40) for 2 hours at 37°C. Staining was stopped by washing three times with PBS followed by 30-min fixation and cryoprotection overnight in 30% sucrose and embedding in cryomatrix for frozen sectioning. Sections were equilibrated at room temperature, washed 3 times with 0.02% NP-40 in PBS and restained for a maximum of 2 hours at 37°C.

### Serum measurement

Blood samples were collected from *Prrx1Cre;Slc38a2* ^*fl/fl*^ and wild type littermate mice through cardiac puncture. Serum P1NP and CTX-I was measured by using P1NP ELISA kit (Immunodiagnostic Systems, AC-33F1) and CTX-I ELISA kit (Immunodiagnostic Systems, AC-02F1) respectively according to the manufacturer’s instructions.

### Cell culture

Primary bone marrow cells were isolated as follows. Briefly, the diaphyses of the femur and tibiae were isolated and all extemporaneous tissue was removed. The epiphyses were removed, and marrow was collected by centrifugation. Red blood cells were lysed, and cells were washed one time before plating. For colony forming efficiency assays, 1 × 10 (Mizoguchi et al., 2014) bone marrow cells were plated in a T25 flask. 3 h after plating, non-adherent cells were removed by washing vigorously with PBS. Cells were then cultured for 7 days under 2% O_2_ before staining with either crystal violent (CFU-F) or alkaline phosphatase for CFU-AP. For CFU-OB assay, colonies were switch to osteogenic media for an additional 7 days before von kossa staining was performed. Colonies with at least 20 cells are counted and quantified. The number of CFU-AP is divided by the number of CFU-F for an individual mouse to give the proportion of CFU-AP positive colonies. For high density cultures, bone marrow cells were plated and cultured under 2% O_2_ for 7 days and washed vigorously after 3 and 6 days to remove non-adherent cells. Cells were trypsinized and passaged at day 7 and replated at a seeding density of 1 × 10 (Bianco and Robey, 2015)cells/mL for all further experiments. Osteoblast differentiation was initiated when cells were confluent by replacing the medium with α-MEM supplemented with 50 mg/ml ascorbic acid and 10 mM β-glycerophosphate for the indicated time with a change of media every 48 h. Mineralization was assessed by alizarin red staining. In the indicated experiments, proline free α-MEM was supplemented with either 0.3 mM L-proline or 5 mM α-methylaminoisobutyric acid (MEAIB) as indicated.

### RNA isolation and qPCR

Total RNA was harvested from cultured cells by scraping the cells in TRIZOL followed by a chloroform extraction. 500ng of total RNA was used for reverse transcription by IScript cDNA synthesis kit (Bio-Rad). SsoAdvanced Universal SYBR Green Supermix (Bio-Rad) was used for qPCR with primers used at 0.1 μ M (listed in Table 1). Technical and biological triplicates were performed in 96-well format on an ABI QuantStudio 6 Pro. The PCR program was set as 95 ℃ for 3min followed by 40 cycles of 95 ℃ for 10s and 60 ℃ for 30s. *Actb* mRNA level was used to normalize expression of genes of interest and relative expression was calculated using the 2^-(ΔΔCt)^ method. PCR efficiency was optimized and melting curve analyses of products were performed to ensure reaction specificity.

**TABLE 1:** RT-qPCR primer sequences.

Gene symbol	Forward	Reverse
*Actb*	AGA​TGT​GGA​TCA​GCA​AGC​AG	GCG​CAA​GTT​AGG​TTT​TGT​CA
*Akp2*	CCA​ACT​CTT​TTG​TGC​CAG​AGA	GGC​TAC​ATT​GGT​GTT​GAG​CTT​TT
*Ibsp*	CAG​AGG​AGG​CAA​GCG​TCA​CT	GCT​GTC​TGG​GTG​CCA​ACA​CT
*Bglap*	CAGCGGCCCTGAGTCTGA	GCC​GGA​GTC​TGT​TCA​CTA​CCT​TA
*Sp7*	CCC​TTC​TCA​AGC​ACC​AAT​GG	AAG​GGT​GGG​TAG​TCA​TTT​GCA​TA

### Western blotting

BMSCs were scraped in RIPA lysis buffer containing cOmplete protease inhibitor and PhosSTOP cocktail tablets (Roche). Following marrow flush, diaphyseal bone was chopped with scissors 100 times in RIPA (50 mM Tris (pH 7.4), 15 mM NaCl, 0.5% NP-40, 0.1% SDS, 0.1% sodium deoxycholate) lysis buffer. Protein concentration was determined by BCA protein assay kit (Thermo). Protein (20 μ g) was loaded on 12% polyacrylamide gel and transferred onto Imuno-Blot PVDF membrane. The membranes were blocked for 1 h at room temperature in 5% milk powder in TBS with 0.1% Tween (TBST) and then incubated at 4°C with the primary antibody overnight. Primary antibodies were used at 1:1000 to detect proteins, listed as follows: Anti-SLC38A2 (RRID:AB_2050321), Anti-Runx2 (RRID:AB_10949892), Anti-ACTB (RRID:AB_330288), Anti-ATUB (RRID:AB_2619646), Anti-Phospho T172 AMPK (RRID:AB_330330) and Anti-AMPK (RRID:AB_330331). Membranes were then incubated at room temperature with Anti-Rabbit IgG (RRID:AB_2099233) or Anti-Mouse IgG, HRP-linked Antibody (RRID:AB_330924) at 1:2000 for 1 h at room temperature. Immunoblots were next developed by enhanced chemiluminescence (Clarity Substrate Kit or SuperSignal West Femto substrate). Each experiment was repeated with at least three independently prepared protein extractions. Densitometry was performed for quantification for each blot.

### Amino acid uptake assay

Amino acid uptake assays were performed as previously described (Shen et al., 2021). Cells were first washed three times with PBS and incubated with Krebs Ringer HEPES (KRH) (120 mM NaCl, 5mM KCl, 2 mM CaCl_2_, 1mM MgCl_2_, 25 mM NaHCO_3_, 5 mM HEPES, 1 mM D-Glucose) with 4 μCi/mL L-[2,3,4-^3^H]-Proline (PerkinElmer NET323250UC), L-[3,4-^3^H]-Glutamine (PerkinElmer NET551250UC), L-[2,3-^3^H]-Alanine (PerkinElmer NET348250UC), L-[^14^C(U)]-Glycine (PerkinElmer NEC276E050UC), or L-[3,4-^3^H]-Glutamate (PerkinElmer NET490001MC) for 5 min at 37°C. Uptake and metabolism were terminated with ice cold KRH and the cells were scraped with 1% SDS. Cell lysates were combined with 8 ml Ultima Gold scintillation cocktail (PerkinElmer 6013329) and CPM was measured using Beckman LS6500 Scintillation counter. For *ex vivo* amino acid uptake assays, the humeri and tibiae were isolated from 4-month-old mice. All extemporaneous tissue and the epiphyses were removed, and the bone marrow was flushed leaving the diaphysis of each bone. Diaphyses were weighed for normalization and the contralateral control bone was incubated at 100°C for 20 min to kill all living cells as a negative control for amino acid adsorption to the bone matrix. Bones were then incubated with KRH containing radiolabeled amino acids for 1 h at 37°C. Uptake and metabolism were subsequently terminated using ice cold KRH. Samples were homogenized in RIPA lysis buffer followed by sonication using an Ultrasonic Processor (VCX130) (Amplitude: 35%, Pulse 1s, Duration: 10 s) and centrifugation. Supernatant from each sample was combined with 8 ml scintillation cocktail and CPM was measured using Beckman LS6500 Scintillation counter. Radioactivity was normalized to CPM from the boiled contralateral controls.

### Flow cytometry

Bone marrow cells were isolated from 4-month-old male and female mice as described above. After red blood cell lysis, the cells were washed with PBS, counted and resuspended at 5 × 10 (Mizoguchi et al., 2014) cells/ml in ice cold PBS containing 2% FBS. 2 μg/ml fluorescent conjugate primary antibodies were added. Antibodies used were anti-CD45-FITC (BD Biosciences, 561088), anti-Sca1-Alexa Fluor 647 (BD Biosciences, 565355), anti-PDGFRα-PE-Cy7 (Thermo, 25-1401-82). Cells were incubated with antibody for 45 min at 4°C. The cells were then washed with PBS 3 times before collection by centrifugation at 400 *g* for 5 min. Cells were resuspended in 500 μL ice cold PBS, 10% FBS, for analysis. EdU incorporation was performed using Click-iT™ EdU Alexa Fluor™ 488 Flow Cytometry Assay Kit. Cells were incubated with EdU (5‐ethynyl‐2′‐deoxyuridine, 10 μM) for 24 h. Cells were then trypsinized, fixed, permeabilized and incubated with Click-iT reaction cocktail for 30 min according to manufacturer’s instructions. Cell viability was analyzed using the Cell Meter™ APC-Annexin V Binding Apoptosis Assay Kit (Cat# 22837). Cells were trypsinized and incubated with APC-Annexin V conjugate and propidium iodide for 30min. For progenitor cell analysis, bone marrow cells were isolated from 4-month-old mice as described above. After red blood cell lysis, the cells were washed with PBS, counted, and resuspended at 5 × 10^6^ cells/mL in ice-cold PBS containing 2% FBS. Fluorescent conjugate primary antibodies (2 μg/ml) were added. The cells were then washed with PBS three times before collection by centrifugation at 400 *g* for 5 min. Cells were resuspended in 500 μL ice-cold PBS and analyzed using FACSCanto II flow cytometer (BD Biosciences). Data were analyzed using FlowJo (v.11).

### Quantification and statistical analysis

Statistical analyses were performed using either Graphpad Prism 6 or R software. One-way ANOVA or unpaired 2-tailed Student’s t-test were used to determine statistical significance as indicated in the text. All data are shown as mean values ± SD. *p* < 0.05 is considered as statistically significant. Sample size (n) and other statistical parameters are included in the figure legends. Most experiments were repeated on 3 independent sets of samples unless noted.

## Results

### SLC38A2 is expressed in osteochondral lineage cells postnatally

**FIGURE 1:**
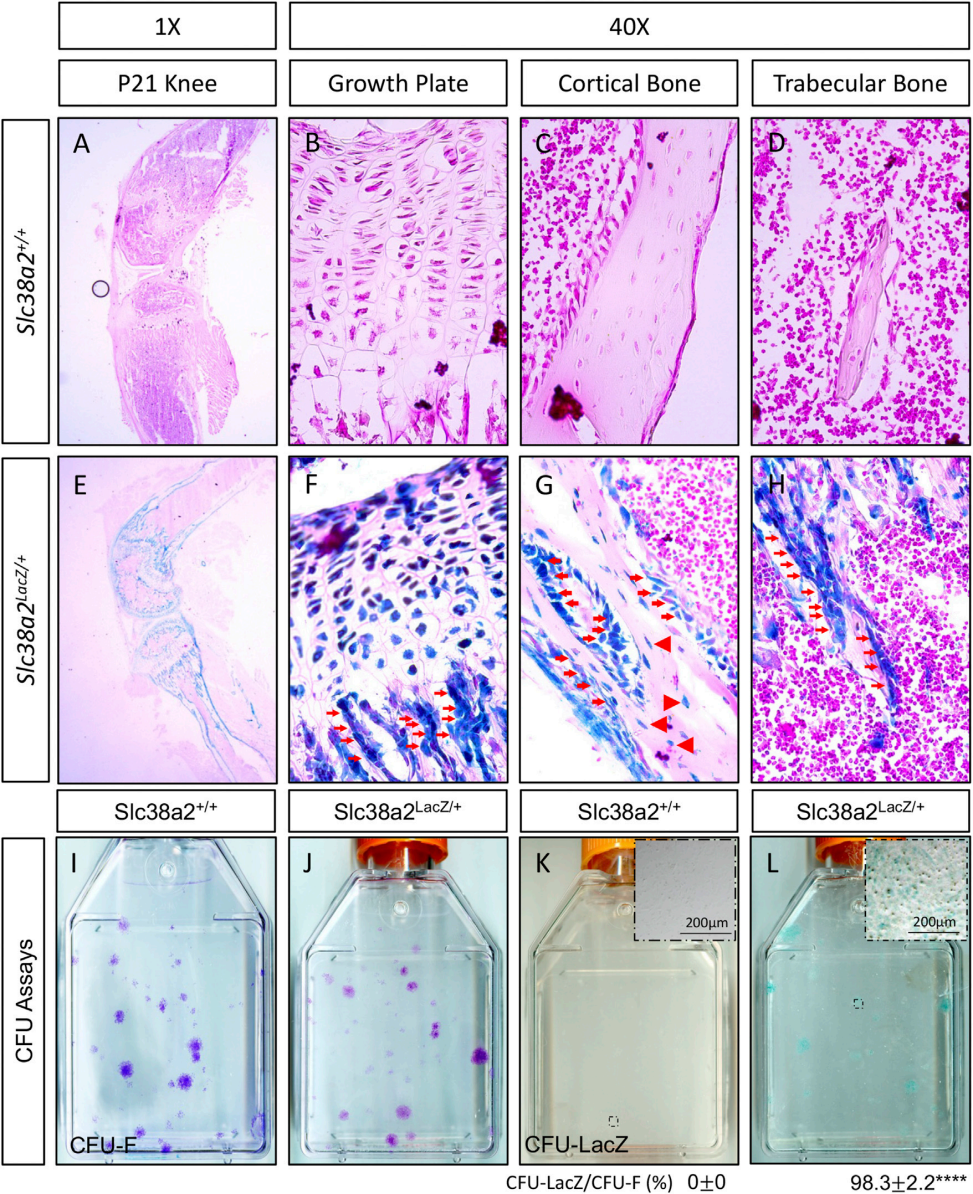
SLC38A2 is expressed in osteoblast lineage cells after parturition. **(A–H)** Postnatal day 21 (p21) *Slc38a2* ^*+/+*^ (wild type) **(A–D)** or *Slc38a2* ^*LacZ/+*^ **(E–H)** knees stained with X-gal [dark blue in **(E–H)**]. LacZ staining is apparent in articular and growth plate chondrocytes, osteoblasts [red arrows in (**G–H**)] and osteocytes [arrowheads in **(G)**]. Note the absence of staining in the wild type mice that don’t express LacZ. Representative images of colony forming unit (CFU) assays stained with Crystal Violet **(I–J)** or X-gal **(K,L)** performed on 2-month-old wild type **(I,K)** or *Slc38a2* ^*LacZ/+*^ **(J,L)** littermates. Note the presence of LacZ expressing colonies in **(L)**. Inset image in **(K–L)** shows high magnification image of individual colony. Quantification of proportion of total colonies that are also LacZ positive shown below. ****p < 0.00005, N = 4 mice.

We previously demonstrated SLC38A2 is a critical regulator of bone development by providing proline (Shen et al., 2022). Unfortunately, nothing is known about SLC38A2 in postnatal bone homeostasis. To eradicate this ignorance, we first sought to determine if SLC38A2 is expressed in bones after parturition. To do this, we utilized mice harboring an in-frame insertion of β-galactosidase (encoded by *LacZ*) into the coding region of *Slc38a2* (*Slc38a2* ^*LacZ*^)*.* β-galactosidase (β-gal) staining demonstrated *Slc38a2* is expressed in both articular and growth plate chondrocytes as well as osteoblast lineage cells at postnatal (p) day p17 (Figure 1). β-gal staining was observed in bone lining cells on both the endosteal and periosteal surfaces (see red arrows) as well as in osteocytes (See red arrowheads in Figure 1). Importantly, we did not observe β-gal staining in wild type littermate controls. Likewise, we did not observe robust β-gal staining in the bone marrow or other tissues (e.g., muscle) of *Slc38a2* ^*LacZ*^ mice (Supplementary Figure S1). Rather, we observed sporadic β-gal staining in individual small cells the bone marrow that may be SSPC or some other unidentified cell type (Supplementary Figure S1). To determine if *Slc38a2* is expressed in SSPC, we performed CFU assays followed by β-gal staining (Figure 1I–L). As expected, no β-gal positive colonies were identified in *Slc38a2* ^*+/+*^ wild type mice (Figure 1K). By comparison, almost all colonies from *Slc38a2* ^*LacZ/+*^ mice (98.3 ± 2.2%) stained positive for β-gal albeit at varying intensities (Figure 1L, see inset image for a weakly stained colony). From these data, we conclude *Slc38a2* is expressed in all stages of the osteoblast differentiation continuum beginning in the SSPC into the osteocyte.

### *Prrx1Cre;Slc38a2* ^*fl/fl*^ mice have reduced bone mass

**FIGURE 2:**
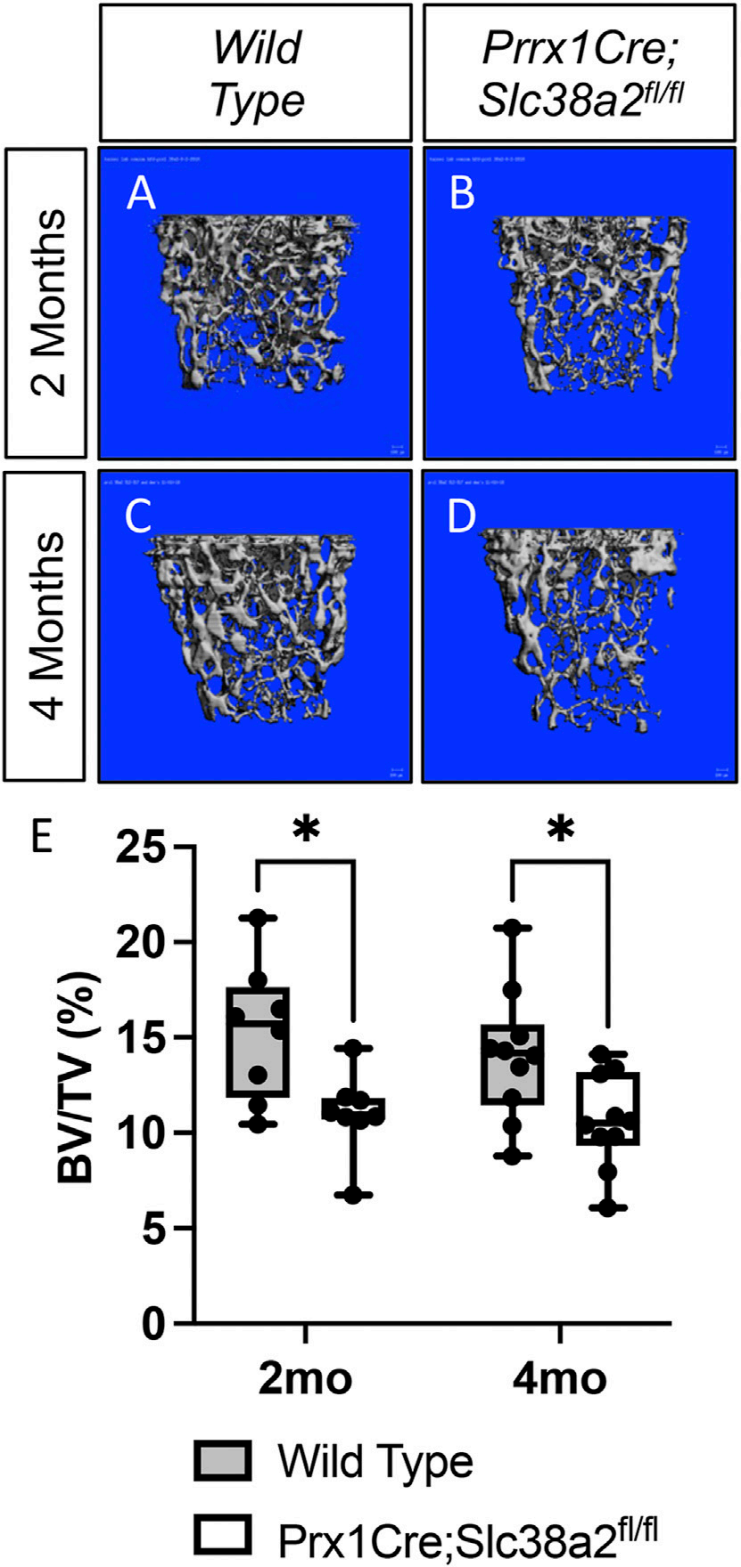
SLC38A2 is required to maintain bone mass postnatally. **(A–D)** Representative µCT images of the trabecular bone in the distal femur of 2- **(A–B)** or 4-month-old **(C–D)** wild type (WT) **(A,C)** or *Prrx1Cre;Slc38a2* ^*fl/fl*^ **(B,D)** male mice. **(E)** Quantification of µCT images. BV/TV—Bone volume/Tissue Volume. *p < 0.005, N = 8 mice each genotype at 2 months. N = 10 for each genotype at 4-months.

**FIGURE 3:**
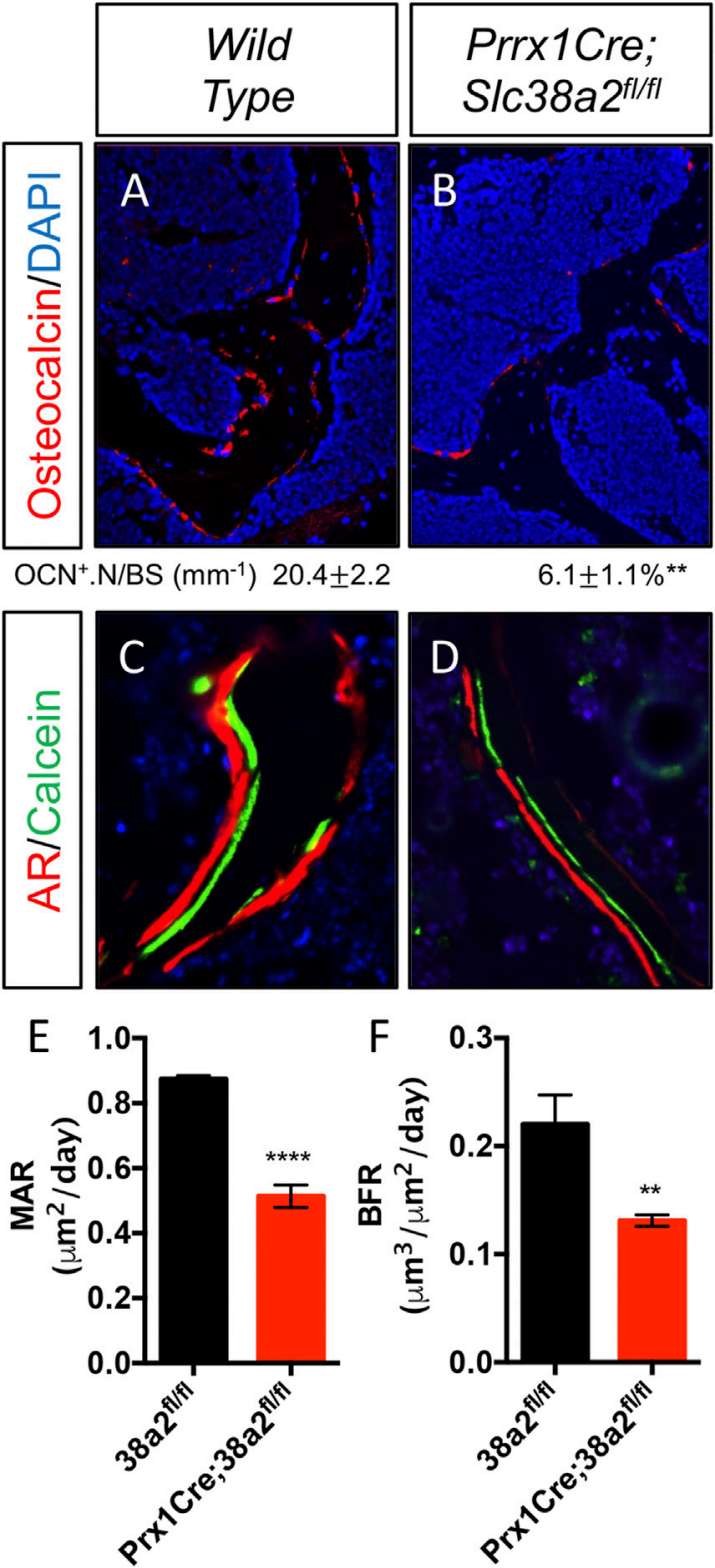
Decreased osteoblast numbers and activity in *Prrx1Cre;Slc38a2* ^*fl/fl*^ mice. **(A–B)** Representative Osteocalcin (OCN) immunofluorescent staining of 4-month-old femur trabecular bone. **(C–D)** Representative calcein and alizarin red double labeled sections of the distal femur from 4-month-old mice. **(E–F)** Quantification of mineral apposition rate (MAR) or bone formation rate (BFR) derived from double labeling. **p < 0.005, ****p < 0.00005, N ≥ 3.

To determine if SLC38A2 is required for postnatal bone homeostasis, we conditionally deleted a floxed allele of *Slc38a2* (*Slc38a2* ^*fl*^) in all stages of osteoblast differentiation using *Prrx1Cre* (Logan et al., 2002). *Prrx1Cre;Slc38a2* ^*fl/fl*^ mice were born at Mendelian ratios and were indistinguishable from wild type littermates (not shown). µCT analyses of 2- and 4-month-old mice found a significant reduction in bone mass in *Prrx1Cre;Slc38a2* ^*fl/fl*^ mutant compared to wildtype littermate controls (Figure 2 and Table 2). Quantification of the µCT analyses demonstrated that *Slc38a2* deletion resulted in significant reduction in trabecular bone volume per tissue volume (BV/TV), trabecular number (Tb.N) and bone mineral density (BMD), while increasing trabecular separation (Tb.Sp) in male mice (Table 2). Cortical bone mass was unaffected (Table 2). By comparison, female *Prrx1Cre;Slc38a2* ^*fl/fl*^ mice only had decreased bone mass at 3 months of age with no detectible difference in bone volume at 2- or 4-months of age (Table 2). Because of this difference, we chose to focus our cellular analyses on male mice due to the more consistent bone phenotype across different timepoints. Histological analyses confirmed the overall reduction in trabecular bone at 4-months of age in these mice (Supplementary Figure S1). We determined the effects of SLC38A2 ablation on osteoblast numbers and bone forming activity using standard and dynamic histomophometry. *Prrx1Cre;Slc38a2* ^*fl/fl*^ mice were characterized by a significant reduction in the overall number of osteocalcin (OCN) positive osteoblasts per bone surface (OCN^+^.N/BS) (Figures 3A,B). Dynamic histomorphometry found *Prrx1Cre;Slc38a2* ^*fl/fl*^ mice had no significant difference in osteoblast coverage as exemplified by the mineralized surface per bone surface (MS/BS) (Table 2). However, the osteoblasts displayed less bone forming activity as both the mineral apposition rate (MAR) and bone formation rate (BFR) were significantly reduced in *Prrx1Cre;Slc38a2* ^*fl/fl*^ mice relative to wild type littermates (Figures 3E–G and Table 2). Consistent with decreased bone formation, serum levels of N-terminal pro-peptide of type 1 collagen (P1NP), a marker of bone formation, was significantly reduced in *Prrx1Cre;Slc38a2* ^*fl/fl*^ mice (4.7 ± 2.1 vs. 2.5 ± 1.5 ng/ml in wild type and *Prrx1Cre;Slc38a2* ^*fl/fl*^ mice respectively, *p* < 0.05 n = 4). TRAP staining identified an modest increase in the number of TRAP positive osteoclasts per bone surface in *Prrx1Cre;Slc38a2* ^*fl/fl*^ mice (9.6 ± 2.3 vs. 15.4 ± 2.9 in wild type and *Prrx1Cre;Slc38a2* ^*fl/fl*^ mice respectively, *p* < 0.05 n = 4) although there were no changes in serum levels of the C-terminal telopeptide of collagen (CTX-1), a marker of bone resorption (21.1 ± 4.2 vs. 20.9 ± 7.2 ng/ml in wild type and *Prrx1Cre;Slc38a2* ^*fl/fl*^ mice respectively, *n* = 4). Thus, SLC38A2 is required to maintain both the number of OCN positive mature osteoblasts and bone matrix production necessary to maintain postnatal bone mass *in vivo*.

**TABLE 2:** Bone Parameters in *Prrx1Cre;Slc38a2* ^*fl/fl*^ mice.

	2 Month		3 Month		4 Month	
	**Slc38a2^fl/fl^**	**Prx1Cre**	**Slc38a2^fl/fl^**	**Prx1Cre**	**Slc38a2^fl/fl^**	**Prx1Cre**
		**Slc38a2^fl/fl^**		**Slc38a2^fl/fl^**		**Slc38a2^fl/fl^**
Male (n)	6	6	N.D.	N.D.	12	11
Weight (g)	26.3 ± 1.4	26.4 ± 2.5	N.D.	N.D.	29.6 ± 3.6	29.1 ± 3.7
BV/TV (%)	16.1 ± 6.6	11.1 ± 2.5^a^	N.D.	N.D.	13.9 ± 3.7	10.6 ± 2.6^a^
Tb.N (1/mm)	5.2 ± 1.1	4.7 ± 0.4	N.D.	N.D.	4.6 ± 0.5	4.2 ± 0.3^a^
Tb.Th (mm)	0.05 ± 0.005	0.04 ± 0.0004^a^	N.D.	N.D.	0.05 ± 0.005	0.04 ± 0.003^a^
Tb.Sp (mm)	0.19 ± 0.04	0.20 ± 0.05^a^	N.D.	N.D.	0.2 ± 0.03	0.22 ± 0.02^a^
Ct.BV/TV	0.55 ± 0.04	0.54 ± 0.02	N.D.	N.D.	0.5 ± 0.02	0.48 ± 0.04
Ct.Th (mm)	0.22 ± 0.02	0.21 ± 0.01	N.D.	N.D.	0.22 ± 0.01	0.21 ± 0.02
T.Ar (mm)	1.60 ± 0.06	1.59 ± 0.02	N.D.	N.D.	1.6 ± 0.1	1.6 ± 0.1
Female (n)	6	6	5	5	5	6
Weight (g)	22.4 ± 2.6	21.9 ± 1.9	24.1 ± 1.9	24.2 ± 1.7	24.3 ± 2.1	24.6 ± 2.8
BV/TV (%)	6.3 ± 2	5.3 ± 1.3	10.7 ± 1.9	6.1 ± 1.4^a^	5.7 ± 2.1	5.6 ± 1.8
Tb.N (1/mm)	3.8 ± 0.3	3.5 ± 0.4	4.2 ± 0.2	3.1 ± 0.2^a^	3.2 ± 0.4	3.3 ± 0.5
Tb.Th (mm)	0.037 ± 0.004	0.038 ± 0.003	0.042 ± 0.002	0.038 ± 0.002^a^	0.039 ± 0.005	0.038 ± 0.003
Tb.Sp (mm)	0.27 ± 0.03	0.29 ± 0.04	0.24 ± 0.01	0.29 ± 0.02^a^	0.32 ± 0.05	0.31 ± 0.05
Ct.BV/TV	0.53 ± 0.02	0.51 ± 0.02	0.55 ± 0.02	0.53 ± 0.02	0.54 ± 0.01	0.51 ± 0.03
Ct.Th (mm)	0.21 ± 0.01	0.20 ± 0.01	0.22 ± 0.01	0.22 ± 0.01	0.24 ± 0.01	0.22 ± 0.02
T.Ar (mm)	1.55 ± 0.03	1.54 ± 0.07	1.61 ± 0.03	1.59 ± 0.01	1.59 ± 0.05	1.58 ± 0.02

a *p* ≤ 0.05 compared to littermate control.

N.D, Not Determined.

### SLC38A2 transports proline to regulate SSPC proliferation

**FIGURE 4:**
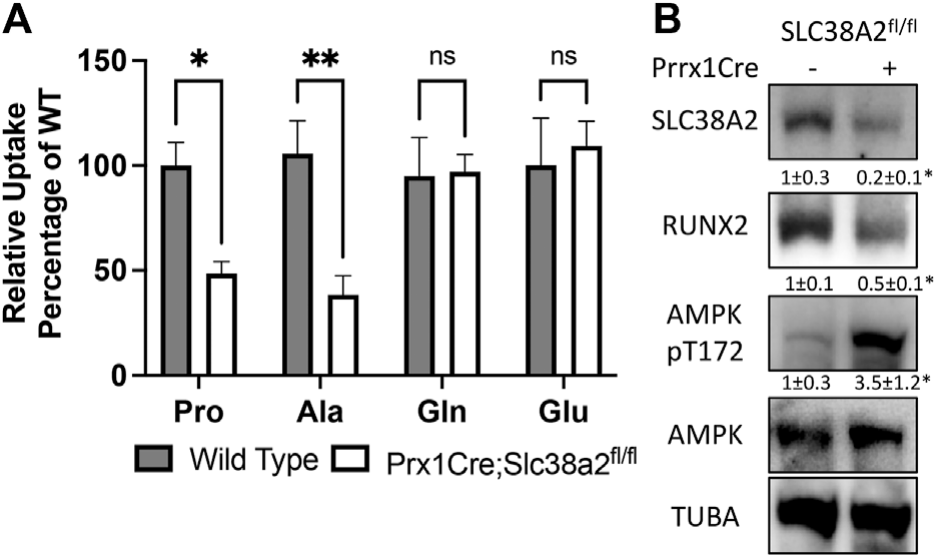
SLC38A2 is the primary transporter of proline and alanine in postnatal bones. **(A)** Radiolabeled amino acid uptake assays in tibial or humeral diaphyses isolated from 4-month-old wild type or *Prrx1Cre;Slc38a2* ^*fl/fl*^ mice. *p < 0.05, **p < 0.005. N = 9, 8, 7 or 5 for Pro, Ala, Gln or Glu respectively. **(B)** Western blot analyses of femur extracts isolated from 4-month-old mice. SLC38A2 and RUNX2 expression normalized to α-tubulin (TUBA), pT172 AMPK normalized to total AMPK. *p < 0.05, N = 3.

We next evaluated amino acid uptake in wild type and *Prrx1Cre;Slc38a2* ^*fl/fl*^ mutant bones using radiolabeled amino acid uptake assays in isolated diaphysis. We observed a significant reduction in both ^3^H-proline and ^3^H-alanine uptake in *Prrx1Cre;Slc38a2* ^*fl/fl*^ mutant mice compared to wild type littermates (Figure 4A). Importantly, the uptake of other known amino acid substrates of SLC38A2 (*e.g.*, ^3^H-glutamine, ^3^H-serine and ^3^H-glycine were not affected by loss of SLC38A2 (Figure 4A and not shown). Likewise, amino acids not known to be transported by SLC38A2 (e.g., ^3^H-glutamate) were also unaffected (Figure 4A). In embryonic osteoblasts, SLC38A2 provides proline to sustain the production of proline enriched osteoblast proteins (Shen et al., 2022). Consistent with reduced proline uptake, *Prrx1Cre;Slc38a2* ^*fl/fl*^ bone extracts were characterized by a significant decrease in the proline enriched protein RUNX2 (10.5% proline) without any change in the expression of a non-proline enriched protein, AMPK (5.2% proline) (Figure 4B). In addition, *Prrx1Cre;Slc38a2* ^*fl/fl*^ bone extracts were characterized by a significant increase in AMPK phosphorylation at T172, a marker of energetic stress. These data indicate SLC38A2 is essential for RUNX2 expression and energetic homeostasis in postnatal bone.

**FIGURE 5:**
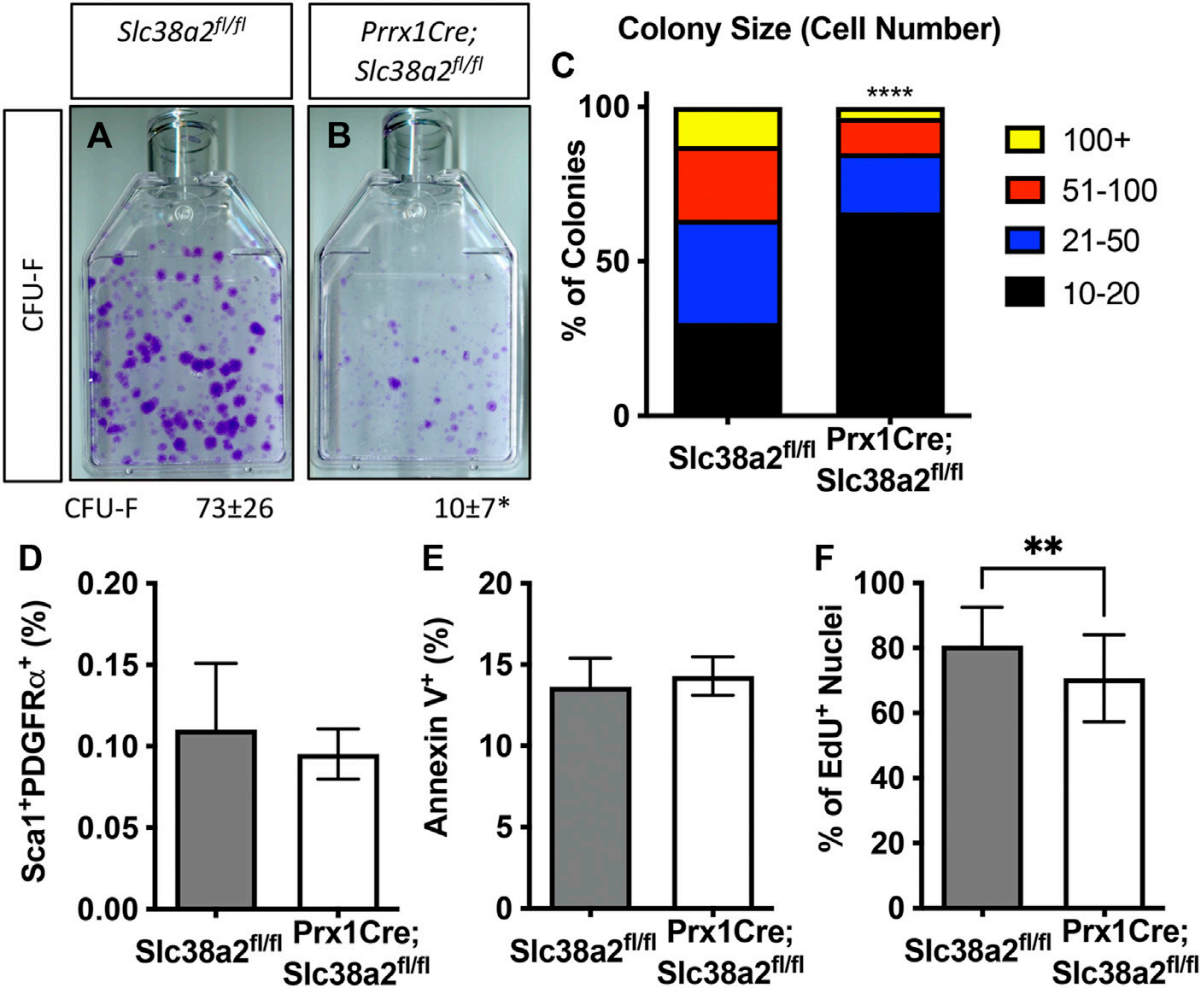
SLC38A2 activity is required for SSPC proliferation. **(A,B)** Representative images of colony forming unit (CFU) assays stained with Crystal Violet. Quantification of the number of colonies containing at least 20 cells is shown below each panel. *p < 0.05 *n* = 5 mice each genotype. **(C)** Prevalence of colonies binned for cell number. ****p < 0.00005. **(D)** Quantification of the CD45^−^, PDGFRa^+^, Sca1^+^ population using flow cytometry. N = 3 mice. **(E, F)** Quantification the number of Annexin V positive cells **(E)** or EdU positive nuclei **(F)**. **p < 0.005, N = 5 mice.

We next evaluated the effects of *Slc38a2* deletion on SSPC by performing colony forming efficiency (CFE) assays in 2-month-old *Slc38a2* ^*fl/fl*^ (Wild type) and *Prrx1Cre;Slc38a2* ^*fl/fl*^ mice. *Prrx1Cre;Slc38a2* ^*fl/fl*^ mice displayed a significant reduction in overall CFE (Figures 5A,B). In addition to reduced CFE, there was a significant bias toward smaller colonies with fewer cells in *Prrx1Cre;Slc38a2* ^*fl/fl*^ mice (Figure 5C). We reasoned this could be due to premature depletion of the SSPC population, reduced proliferation or increased apoptosis. SSPC are enriched in the CD45^−^PDGFRα^+^Sca-1^+^ bone marrow fraction (Morikawa et al., 2009; Omatsu et al., 2010; Park et al., 2012). Direct analysis of this bone marrow population using flow cytometry demonstrated no difference in the overall number of CD45^−^PDGFRα^+^Sca-1^+^ SSPC in *Prrx1Cre;Slc38a2* ^*fl/fl*^ mice (Figure 5D). This indicates the CFE defect is not the result of a premature loss of the SSPC population. We next evaluated the effect of *Slc38a2* ablation on both cell death and proliferation. Reduced CFE in *Prrx1Cre;Slc38a2* ^*fl/fl*^ mice was not the result of increased apoptosis as there was no difference in the number of Annexin 5 positive cells isolated from *Prrx1Cre;Slc38a2* ^*fl/fl*^ mice compared to wild type littermates (Figure 5E). Rather, cells isolated from *Prrx1Cre;Slc38a2* ^*fl/fl*^ mice incorporated significantly less EdU compared to wild type littermates (Figure 5F).

**FIGURE 6:**
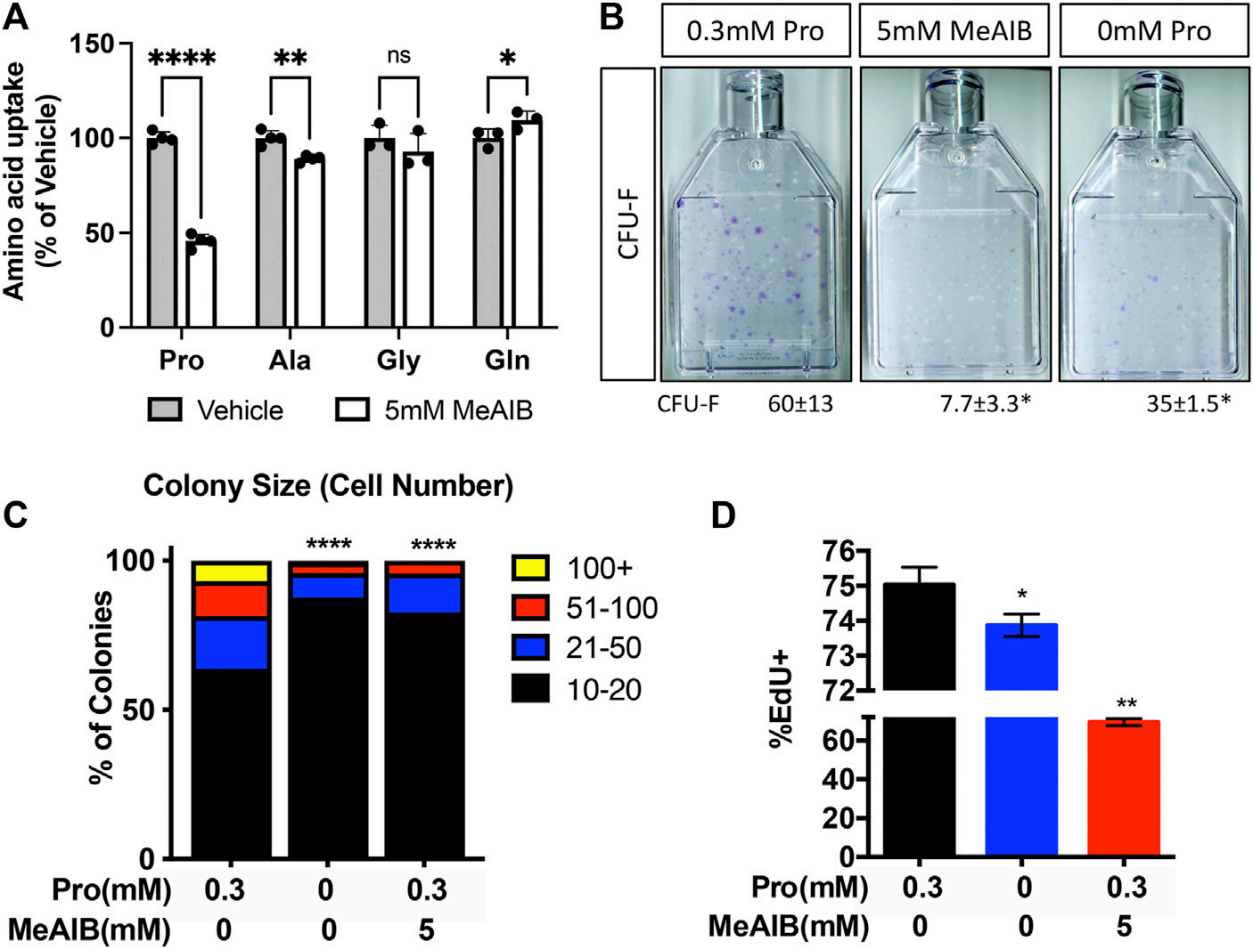
Proline and alanine uptake are required for SSPC proliferation. **(A)** Effect of MeAIB on radiolabeled amino acid uptake in high density BMSC isolated from 2-month-old C57Bl6/J mice. *p < 0.05, **p < 0.005. ****p < 0.00005. N ≥ 3 **(B)** Representative images of the effect of MeAIB or proline withdrawal on CFU-F. Quantification of the number of colonies containing at least 20 cells is shown below each panel. N = 4 mice each treatment. **(C)** Prevalence of colonies binned for cell number. **(D)** Effect of MeAIB or proline withdrawal on EdU incorporation in bone marrow cells plated at clonal density. *p < 0.05, **p < 0.005, ****p < 0.00005.

To determine if decreased proliferation was directly or secondarily related to the loss of SLC38A2, we acutely inhibited SLC38A2 in wild type BMSC using α-methylaminoisobutyric acid (MeAIB). MeAIB significantly reduced the uptake of proline by over 50% and alanine to a lesser extent in BMSC (Figure 6A). MeAIB did not affect glycine uptake and actually increased glutamine uptake (Figure 6A). Increased glutamine uptake is likely compensatory due to increase glutamine dependent proline synthesis as we previously published (Shen et al., 2022). To determine if proline uptake is essential for SSPC proliferation, we performed CFE assays from C57Bl/6 mice and cultured these colonies in replete media containing either 5 mM MeAIB to acutely inhibit proline uptake or in media lacking proline. Reducing proline uptake using MeAIB significantly reduced CFE and decreased the number of cells per colony in wild type cells (Figures 6B,C). Likewise, culturing cells in proline free media reduced both CFE and the number of cells per colony albeit to a lesser extent compared to MeAIB treatment (Figures 6B,C). These data indicate that SLC38A2 ablation does not affect overall SSPC numbers or viability but likely governs SSPC proliferation. To test this hypothesis directly, we cultured BMSC isolated from C57BL/6J mice at clonal density in media containing either 5 mM MeAIB or 0 mM proline and evaluated EdU incorporation. Inhibiting proline uptake in these ways inhibited BMSC proliferation as shown by a statistically significant reduction in EdU incorporation (Figure 6D). Importantly, MeAIB had a more profound impact on proliferation likely due to reduced proline and alanine uptake. Thus, our data indicate that SLC38A2 provides SSPC with proline and alanine necessary for robust SSPC proliferation.

**FIGURE 7:**
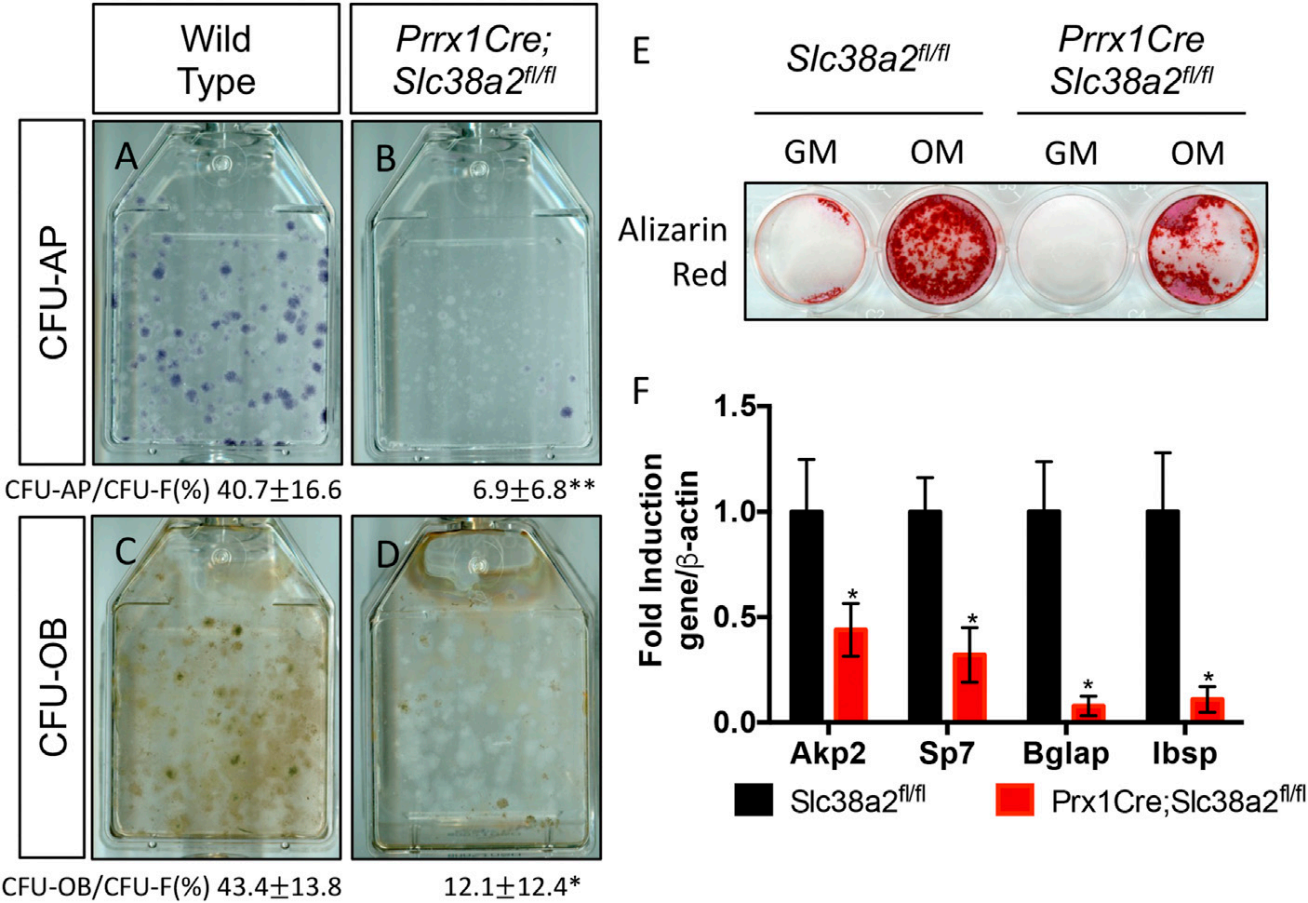
SLC38A2 ablation reduces osteogenic differentiation and matrix mineralization *in vitro*. **(A–D)** Representative images of CFU assays stained with alkaline phosphatase (CFU-AP) or von kossa (CFU-OB) from wild type **(A,C)** or *Prrx1Cre;Slc38a2* ^*fl/fl*^ mice **(B,D)**. Quantification of the proportion of colonies containing at least 20 stained cells divided by the total number of colonies (CFU-F, from Figure 5) shown below each panel. *p < 0.05, n = 5 mice each genotype. **(E–F)** Evaluation of osteoblast differentiation in primary BMSC as measured by Alizarin red staining **(E)** or osteogenic gene expression **(F)** measured by RT-qPCR. BMSC were cultured in growth media (GM) or osteogenic media (OM). Gene expression normalized to *Actb* (β-actin) mRNA levels. *p < 0.05. N = 3 individual mice.

Finally, since SLC38A2 is essential for RUNX2 expression (Figure 4B) we sought to understand if SLC38A2 ablation affected osteoblast differentiation. To do this, we performed CFE assays and stained the colonies for alkaline phosphatase (AP) expression to evaluate osteoblast potential. Fewer colonies from *Prrx1Cre;Slc38a2* ^*fl/fl*^ mice had AP staining indicating they are less osteogenic compared to wild type colonies (Figures 7A,B). To test if SLC38A2 affected osteoblast differentiation, we treated established colonies from wild type and *Prx1Cre;Slc38a2* ^*fl/fl*^ mice with osteogenic media for an additional 7 days and evaluated mineralization using Von Kossa staining. This found a significant reduction in the proportion of Von Kossa-stained osteoblastic colonies (CFU-Ob) from *Prrx1Cre;Slc38a2* ^*fl/fl*^ mice compared to wild type littermates (Figures 7C,D). Likewise, BMSC isolated from *Prrx1Cre;Slc38a2* ^*fl/fl*^ mice displayed diminished matrix mineralization as well as reduced osteoblast marker gene expression (e.g., *Akp2*, *Sp7*, *Ibsp* and *Bglap*) (Figures 7E,F). Thus, SLC38A2 is essential for osteoblast differentiation.

## Discussion

Here we present data demonstrating that SLC38A2 is a critical regulator of postnatal bone mass in mice. Genetically ablating SLC38A2 in mesenchymal progenitors and their derivatives using *Prrx1Cre* results in decreased bone mass in both male and female mice. The bone phenotype was more pronounced in male mice with decreased bone mass observed at both 2- and 4-months of age. By comparison, decreased bone mass was only apparent in female mice at 3-months of age. Mechanistically, decreased bone mass is attributed to multiple factors: first, a reduction in overall osteoblast numbers due to decreased SSPC proliferation and reduced osteoblast differentiation in SLC38A2 deficient cells. Second, SLC38A2 deficient osteoblasts have significantly reduced bone matrix production. Collectively, this results in significantly fewer active osteoblasts and declining bone mass with age. Finally, SLC38A2 deficient mice have increased osteoclast numbers which likely exacerbates the low bone mass phenotype over time.

SLC38A2 is a system A transporter which mediates the sodium dependent unidirectional import of proline, alanine, serine, glycine and glutamine in different cellular contexts (Bröer et al., 2016; Morotti et al., 2019). In the context of adult bone, we found that SLC38A2 transports proline and alanine. Our data indicates SLC38A2 is the primary proline and alanine transporter in adult bone, mediating 50% of proline and ∼55% of alanine uptake. It is interesting to note that SLC38A2 ablation in the embryo specifically reduced proline uptake without affecting the uptake of alanine or any other amino acid (Shen et al., 2022). This discrepancy may reflect innate differences in intramembranous and endochondral bones, different metabolic needs associated with bone ;development versus homeostasis or distinct metabolic requirements at different stages in the osteochondral lineage. Regardless, it is not clear how SLC38A2 substrate specificity is regulated and what the importance of this difference is for bone mass. Considering these findings, a better understanding of the molecular regulation of SLC38A2 activity and substrate specificity is warranted.

SLC38A2 dependent proline and alanine uptake likely affects SSPC proliferation and osteoblast differentiation in multiple ways. First, osteoblasts require proline to support the synthesis of proline enriched osteoblast proteins like RUNX2 to promote osteoblast differentiation and bone formation. Indeed, RUNX2 was significantly reduced in bone extracts from *Prrx1Cre;Slc38a2* ^*fl/fl*^ mice (Figure 3B). It is important to note that RUNX2 is also essential for osteoblast progenitor proliferation by regulating FGFR2 and FGFR3 expression (Kawane et al., 2018). In addition to direct incorporation into protein, proline can play important roles in carbon and nitrogen metabolism, bioenergetics, oxidative stress protection, cell signaling, nutrient adaptation and cell survival (Liu et al., 2012a; Liu et al., 2012b; Phang et al., 2012; Nagano et al., 2017; Hollinshead et al., 2018; Phang, 2019). For example, proline can be oxidized in the inner mitochondrial membrane to form pyrroline-5-carboxylate (P5C) by proline dehydrogenase (PRODH). PRODH is a flavin dinucleotide (FAD) dependent enzyme that donates electrons to complex II of the electron transport chain coupling proline oxidation to ATP synthesis (Liu et al., 2012b; Phang et al., 2012; Elia et al., 2017; Olivares et al., 2017). P5C can be converted back into proline by the NADPH dependent enzyme pyrroline-5-carboxylate reductase (PYCR) in the proline cycle or can be converted into glutamate or other intermediate metabolites. While a role for proline oxidation during SSPC proliferation and osteoblast differentiation has not been described, *Prxx1Cre;Slc38a2* ^*fl/fl*^ bone extracts had increased AMPK pT172, a marker of energetic stress. This could be due to reduced proline oxidation or altered glucose flux to support alanine and proline biosynthesis to compensate for loss of SLC38A2 activity. Regardless, these data highlight a new regulation of osteoblast energetics by SLC38A2.

Alanine is similarly required for proliferation in various cellular contexts. Pancreatic ductal adenocarcinoma cells upregulate SLC38A2 to increase alanine uptake and fuel proliferation (Parker et al., 2020). The role of alanine in these processes is not well defined but likely centers on the regulation of protein synthesis. For example, T lymphocytes use alanine primarily for protein synthesis to support proliferation and cell growth (Ron-Harel et al., 2019). In the context of osteoblasts, little is known about the role of alanine uptake to support protein synthesis, proliferation or differentiation. Neonatal calvarial osteoblasts actively synthesize alanine from glutamine and asparagine to support protein synthesis (Sharma et al., 2021). Indeed, reducing alanine biosynthesis by inhibiting glutamine uptake or metabolism was associated with reduced proliferation in both SSPC and preosteoblasts (Yu et al., 2019; Sharma et al., 2021). Decreased proliferation is likely due to many factors in these glutamine depletion models; however, it is intriguing to speculate that alanine depletion contributes to these phenotypes. Regardless, it will be important to define the role and necessity of alanine uptake and biosynthesis for SSPC proliferation, osteoblast differentiation and bone formation.

We observed increased osteoclast numbers in *Prxx1Cre;Slc38a2* ^*fl/fl*^ mice. This likely contributes to decreasing bone mass in *Prxx1Cre;Slc38a2* ^*fl/fl*^ mice. The molecular basis for this is unclear but likely involves a non-cell autonomous mechanism as *Prrx1Cre* is not active in osteoclasts or monocyte/macrophage progenitors. Osteoclast activity may be a directly influenced by the energetic or synthetic needs of osteoblasts. Collagen and bone matrix proteins may be thought of as a major source of proline or other amino acids that can be made available in times of nutrient depletion. Indeed, collagen serves as a proline reservoir for pancreatic ductal adenocarcinoma cells to use when other nutrients are limited (Olivares et al., 2017). It is intriguing to speculate that osteoclastic hydrolysis of collagen and other bone matrix proteins functions to provide proline or other amino acids to osteoblasts in order to assuage amino acid needs associated with osteoblast differentiation and bone formation. In this case, SLC38A2 deficient osteoblasts have a proline and alanine deficit and secondarily stimulate osteoclast differentiation and activity to provide proline.

Our data indicates that proline uptake in fetal and adult bones is mediated primarily by SLC38A2 (this work and (Shen et al., 2022)). Previous reports noted differences in proline uptake characteristics between embryonic and adult bone suggesting the proline transport systems change throughout life. For example, sodium withdrawal inhibited proline uptake by more than 90% in fetal rat calvariae but only reduced proline uptake by approximately 40–50% in tibial diaphyses from 6-week-old rats (Finerman and Rosenberg, 1966; Hahn et al., 1969; Yee, 1988). Consistent with these studies, we found SLC38A2 mediates approximately 50% of proline uptake in tibial diaphyses from 4-month-old mice (Figure 4B). The share of proline uptake was similar to neonatal calvarial cells where SLC38A2 mediates approximately 60% of proline uptake (Shen et al., 2022). These data demonstrate SLC38A2 dependent proline uptake is consistent at embryonic and postnatal time points. Thus, it is likely the transport systems mediating SLC38A2 independent proline uptake differ throughout life. It is important to note that we did not evaluate the expression or activity of other putative proline transporters, nor did we define the transport characteristics of proline uptake in either embryonic or adult bone. It will be important to identify the transporters mediating SLC38A2 independent proline uptake as well as to understand their function during osteoblast differentiation and bone development.

While *Slc38a2* deletion equally perturbed bone development in both sexes, *Slc38a2* deletion more strongly and consistently affected bone mass in male mice. This sexually dimorphic effect was unexpected and the molecular basis for this discrepancy is presently unclear. This may represent a differential requirement for SLC38A2 in bone homeostasis in males and females. Consistent with this, male bones have higher SLC38A2 protein expression compared to female bones. Alternatively, the dimorphic effect may be due to hormonal regulation as estrogen suppresses System A dependent proline uptake in breast cancer cells (Hissin and Hilf, 1979; Bhat and Vadgama, 2002; Velazquez-Villegas et al., 2014). Thus, female mice may have depressed osteoblast differentiation and bone formation because of estrogen on proline uptake. Future studies are warranted to understand the temporal and sex specific requirements of SNAT2 dependent proline uptake during bone homeostasis.

## Data Availability

The original contributions presented in the study are included in the article/Supplementary Material, further inquiries can be directed to the corresponding author.

## Supplementary material

Supplementary Figure S1 Evaluation of *Slc38a2* expression. **(A)** Low magnification image of postnatal day 21 (p21) *Slc38a2* ^*LacZ/+*^ knee joint stained with X-gal. Boxed regions shown in **(B–D)**. In the marrow **(B)** LacZ staining is apparent in isolated cells (arrows). In muscle, LacZ staining is apparent in vessels **(C,D)**.

Supplementary Figure S2 Histological characterization of *Prrx1Cre;Slc38a2* ^*fl/fl*^ bones. **(A,B)** Representative hematoxylin and eosin staining of 4-month-old distal femur trabecular bone. **(C,D)** Representative tartrate resistant acid phosphatase (TRAP) stained sections of the distal femur from 4-month-old mice. Boxed regions shown at higher magnification in **(E,F)**. *p < 0.05, N = 4.
