# Supplementary figures and images for: SLC38A2 provides proline and alanine to regulate postnatal bone mass accrual in mice
Source: Front Physiol. 2022 Sep 23;13:992679. doi: 10.3389/fphys.2022.992679 (PMC9538353; doi:10.3389/fphys.2022.992679)

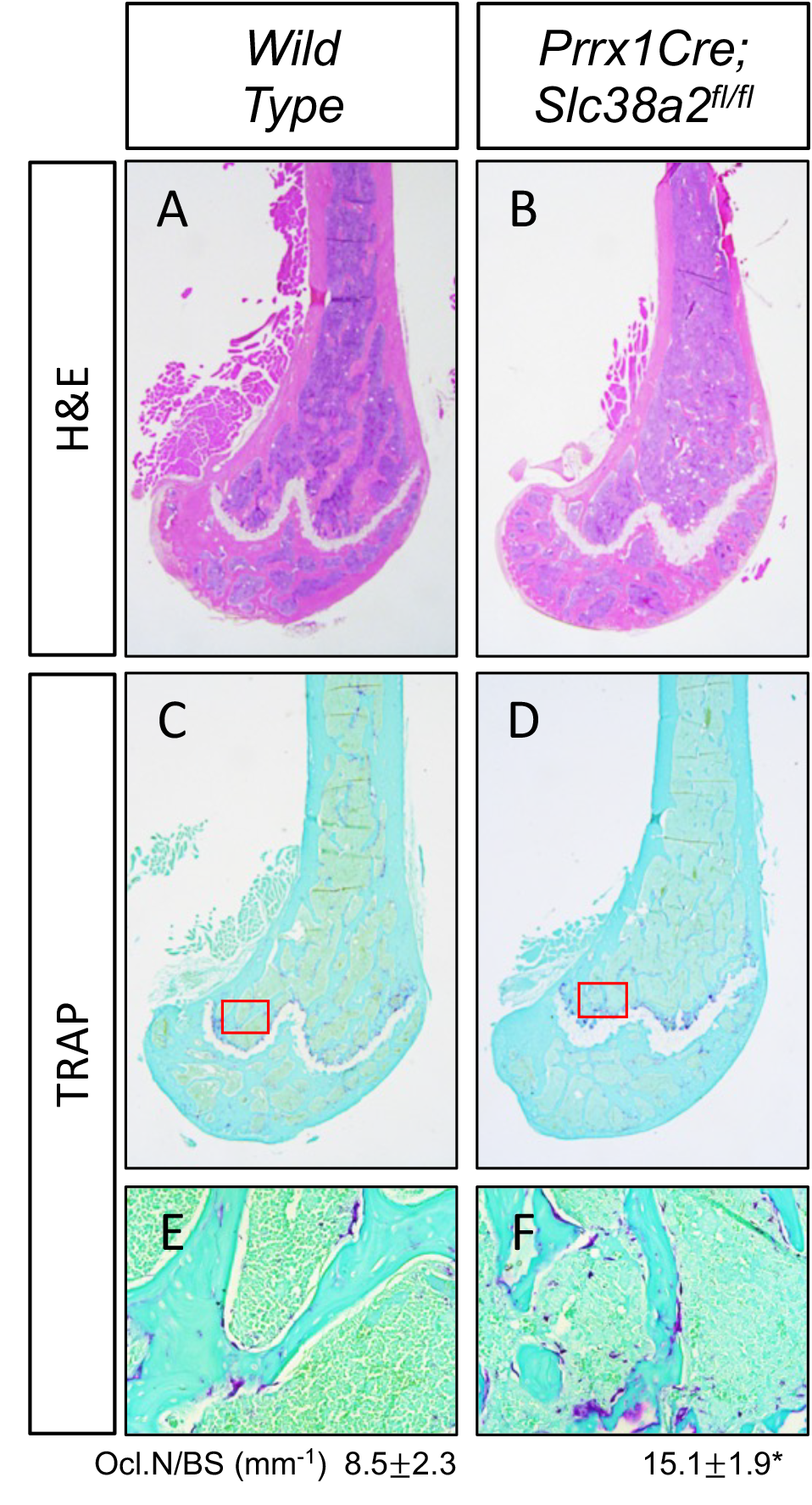

Supplement: Supplementary file 1 [file Image2.TIF]

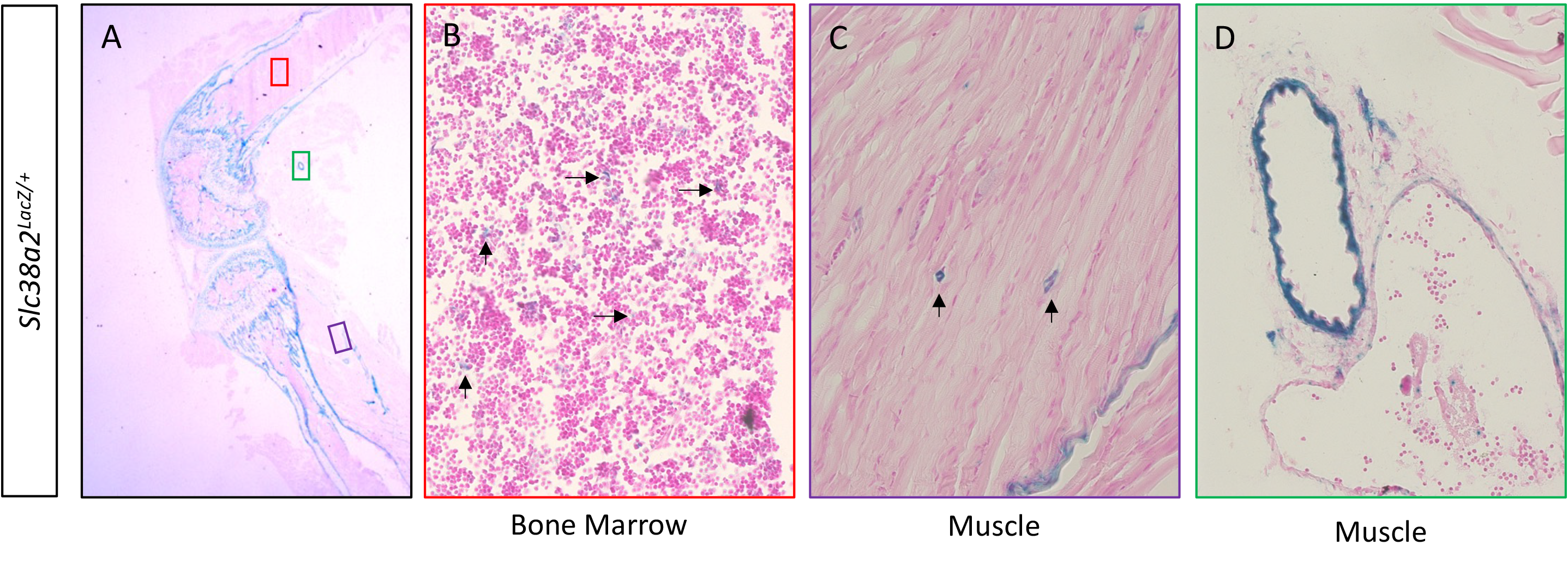

Supplement: Supplementary file 2 [file Image1.TIF]
